# Segmentation and Classification Approaches of Clinically Relevant Curvilinear Structures: A Review

**DOI:** 10.1007/s10916-023-01927-2

**Published:** 2023-03-27

**Authors:** Rajitha KV, Keerthana Prasad, Prakash Peralam Yegneswaran

**Affiliations:** 1 Department of Biomedical Engineering, Manipal Institute of Technology, Manipal Academy of Higher Education, Manipal, 576104 Karnataka India; 2 Manipal School of Information Sciences, Manipal Academy of Higher Education, Manipal, 576104 Karnataka India; 3 Department of Microbiology, Kasturba Medical College, Manipal Academy of Higher Education, Manipal, 576104 Karnataka India

**Keywords:** Curvilinear structures, Fungal hyphae, Corneal nerves, Retinal vessels, Segmentation, Deep learning

## Abstract

Detection of curvilinear structures from microscopic images, which help the clinicians to make an unambiguous diagnosis is assuming paramount importance in recent clinical practice. Appearance and size of dermatophytic hyphae, keratitic fungi, corneal and retinal vessels vary widely making their automated detection cumbersome. Automated deep learning methods, endowed with superior self-learning capacity, have superseded the traditional machine learning methods, especially in complex images with challenging background. Automatic feature learning ability using large input data with better generalization and recognition capability, but devoid of human interference and excessive pre-processing, is highly beneficial in the above context. Varied attempts have been made by researchers to overcome challenges such as thin vessels, bifurcations and obstructive lesions in retinal vessel detection as revealed through several publications reviewed here. Revelations of diabetic neuropathic complications such as tortuosity, changes in the density and angles of the corneal fibers have been successfully sorted in many publications reviewed here. Since artifacts complicate the images and affect the quality of analysis, methods addressing these challenges have been described. Traditional and deep learning methods, that have been adapted and published between 2015 and 2021 covering retinal vessels, corneal nerves and filamentous fungi have been summarized in this review. We find several novel and meritorious ideas and techniques being put to use in the case of retinal vessel segmentation and classification, which by way of cross-domain adaptation can be utilized in the case of corneal and filamentous fungi also, making suitable adaptations to the challenges to be addressed.

## Introduction

Clinicians nowadays, depend upon laboratory reports including microscopic examination and image analysis to confirm diagnosis of skin infections and keratitis caused by fungi, ocular diseases involving cornea and retina. Recently, analysis of corneal nerves affected by density changes and curvature abnormalities has attracted special attention in diabetic clinics [1]. Segmentation of curvilinear structures such as retinal vessels, corneal nerves and fungal filaments has gained due attention in the last few years evidenced by the plethora of publications dealing with conventional and deep learning (DL) techniques in this area. Curvilinear structures have long, thin and line-like features with marked pixel-intensity difference from those of their neighbors [2]. Appearance and sizes of these structures vary widely making the analysis tedious. Manual analysis of even high quality data acquired through the advanced technologies is cumbersome and time consuming. Thus the need for efficient and robust automation methods for reconstruction of these structures cannot be overemphasized [3]. Though the traditional machine learning (ML) techniques for segmentation performed well [4], ability for both generalization and automatic feature learning was found to be a limitation [5]. An upsurge of DL studies in automation of medical image analysis in recent times using large input data avoided human interference, but at the same time provided good generalization and recognition capability. Deep convolutional neural network (CNN) is known to learn features of the training images with various filters applied at each layer. Classification and associated learning are then effected by providing required additional layers [6]. DL has emerged as a research trend in the upgradation of ML with the help of artificial intelligence wherein the final segmentation result can be obtained once the input image is fed [7].

The goal of this review is to give an update of the advancements that have taken place, since 2015, in traditional ML and DL techniques pertaining to the aforementioned major disease areas. These methods addressed the different challenges that come across in the detection of curvilinear structures. Studies published in the first and second quartile journals and conference proceedings have been considered as the basis for reference. Several reviews have appeared on retinal blood vessels and corneal nerves separately. A comprehensive review covering retinal blood vessel, corneal nerves and fungal structures with emphasis on medical diagnosis is lacking. This is an attempt to unify developments in segmentation/classification of microscopic images of the three curvilinear structures.

Organization of this paper is as given below. An overview of the current state-of-the-art segmentation and classification techniques for retinal, corneal and fungal images collected from the literature reported in the period between 2015 to 2021, is provided in sections "Retinal Blood Vessels", "Corneal Nerve Images" and "Filamentous Fungi" respectively. It has been sourced from PubMed, Web of Science, IEEE Xplore, and Google Scholar. In section "Discussion", we discussed the highlights of different approaches adopted and also presented our thoughts on the performance of methods reviewed. In section "Conclusion", is included our conclusive remarks on the scope for further investigations in the retinal, corneal and fungal categories.

## Retinal blood vessels

Structural changes taking place in retinal blood vessels as a result of diabetic retinopathy [8], glaucoma [9], age-related macular degeneration [9], hypertension [10], stroke [11] and cardiovascular diseases [10] call for immediate medical attention so that remedial measures can be effected at the earliest to prevent permanent retinal damage. Several attempts have been made to devise methods for automating vessel segmentation without compromising accuracy, speed and reproducibility [12].

**Fig. 1:**
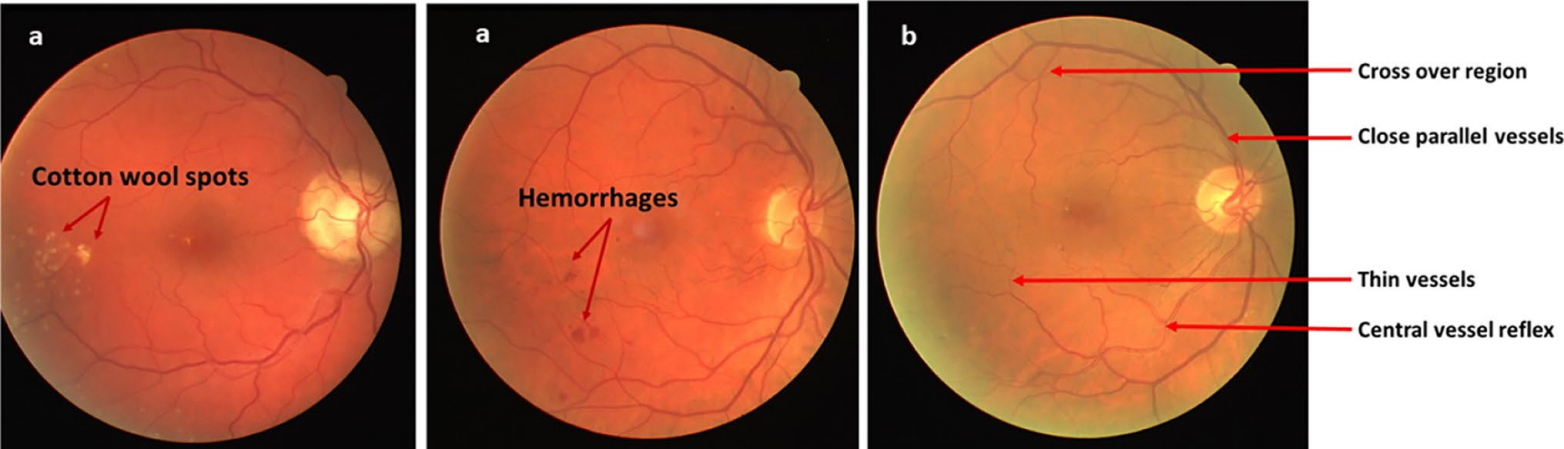
Retinal fundus images (from DRIVE) indicating **a** Artifacts **b** Other challenging features

The following are the major challenges which arise in automation of segmentation of retinal vessels. These are also depicted in Fig. 1.

- Central vessel reflex (CVR): A vessel appearing as two split ones due to light reflex.
- Chances of missing thin vessels in the segmentation map due to poor contrast.
- Fused appearance in segmentation for two close by vessels.
- Broken vessels at bifurcations/crossover points (BC).
- Artifacts such as noise, illumination differences, blur, hemorrhages and cotton wool spots which can delude precise segmentation.

Efforts in the direction of sorting out these challenges are summarized in the following sections "Segmentation Techniques" and "Arteriovenous Classification", wherein segmentation techniques and artery/vein (A/V) classification of retinal vessels are presented. Only DRIVE dataset has been considered for indicating performance metrics in the case of retinal blood vessels.

### Segmentation techniques

Both traditional and DL approaches are discussed in sections "Traditional Methods" and "Deep Learning Methods" respectively.

#### Traditional methods

In spite of the several methods reported under ML, there is relevance and logic in reviewing traditional techniques, as these can still be the components of the newer methods. Methods based on template matched filtering, morphology, vessel tracing/tracking and multiscale techniques come under this category.

Matched filters (MF) which are optimal linear types, are meant to maximize signal to noise ratio. This approach compared the intensity variations of the cross-section profile of the retinal image with the predetermined template, following which suitable thresholding techniques were adopted [13]. Prior knowledge of the primary user signal (template) is essential for its operation. Kar et al. [14–16] suggested three techniques. Two of these, used matched filtering with Laplacian of Gaussian (LoG), followed by area thresholding; however, the pre-processing steps were different; one used the curvelet-based edge enhancement and the other involved contrast limited adaptive histogram equalization (CLAHE) and curvelet transform. The third method employed MF followed by fuzzy conditional entropy. Pre-processing was carried out by curvelet transform. Certain other MF-utilised vessel enhancement methods consisted of 2D Gabor filter [17, 18], Gumbel probability density function (PDF) based filter [19], combination of shifted filter responses (B-COSFIRE) [20] and anisotropic diffusion filter [21]. Suitable thresholding techniques followed, except in the method suggested by Liu et al. [20], which employed support vector machine (SVM) for segmentation with the help of contrast and diffusion maps. Singh and Srivastava [19] adopted skewed Gumbel PDF method to match the non-Gaussian gray scale cross-section distribution profile of retinal images. Yet another approach suggested by Rezaee et al. [22] consisted of skeletonization and threshold selection as computed from fuzzy entropy. Kovacs and Hajdu [23] used a generalized Gabor function-based template to extract the centerline of vessels. Subsequently, intensity pattern of vessel contours gathered through training databases were reconstructed. It facilitated a more accurate modeling of intensity distribution compared with conventional Gabor filter.

Morphology based methods consisted of simple operations related to image shapes. Here, the defined structuring element was assumed to be linear. Twisted parts of the vessel could cause limitations in this method. In order to distinguish vasculature from pathological lesions, Imani et al. [24] employed morphological component analysis and Morlet wavelet transform. Dash and Bhoi, [25] used mean-C thresholding followed by morphological cleaning so as to discard the disjoint regions. Hassan et al. [26] used a hybrid of adaptive thresholding for pre-processing and Hidden Markov model to predict pixels belonging to vessels. Post-processing using morphological steps could remove unwanted isolated pixels and avoid discontinuities caused by occlusion in the segmented vessel images, giving a sensitivity of 81% and specificity of 97%. Jiang et al. [27] made use of top-hat transform to highlight the retinal vessels and smoothen the non-vessel background. Global thresholding was used for binary classification. A method different from ‘thresholding’, more adaptable to thin capillaries, was that of a first-order derivative filter. Results of both the venule and capillary phases when overlapped could detect the vasculature with abundant thin vessels. The overall sensitivity and specificity values were 84% and 97% respectively.

Yet another technique consisted of vessel tracing and tracking which begins with a seed point. The local information was used as a guide to provide vessel widths and connectivity information at bifurcation and crossover (BC) points. Nergiz and Akın [28] aiming at solving obstructive lesion (OL) challenges in vessel segmentation, conducted a study which could be categorised as kernel-based approach, since it used Frangi vesselness filter and also structure tensor. The former enhanced the vessel-like structures while suppressing the non-vascular structures using Hessian based approach. The structure tensor is a matrix representation which highlights the edge and corner information. It was applied to the response of the Frangi filter. Otsu thresholding and tensor colouring for better visualization were the other steps which followed. Sensitivity and specificity recorded were 81% and 93.4% respectively.

Finally, multiscale (MS) methods which easily detected vessels of different thickness [29] were also used so as to mimic human visual functioning mechanism. Objects at both low and high spatial frequencies could be detected by processing the images at different scales. One method for doing this was by altering the image size, for example, using Gaussian pyramid; yet another involved changing the filter size. Thick vessels were detected best even at low spatial scales, whereas thin vessels could be detected only in high spatial scales. Out of the many MS processing methods published in the period under review, clustering of methods which could address similar challenges, was more appealing.

In tackling OL, pixel-wise directional process, MS matched filter and gray scale bottom hat transform were put to use by Lazar and Hadju [30]. Region growing and k-nearest neighbor (KNN) completed the post-processing part. In a different approach, second-order Gaussian derivatives, wavelet, pixel, and Gaussian scale-space features were fed to the random forest (RF) classifier by Zhang et al.[31]. Thresholding technique has been used in the following methods. Hessian eigenvalue analysis followed by percentile-based thresholding was the approach adopted by Annunziata et al. [32]. BahadarKhan et al. [33] applied the same Hessian eigen value analysis, followed by Otsu thresholding. Multilevel thresholding was also used to perform segmentation, where automatic selection of optimal n-level threshold was a problem. Hassan and Hassanien [34] sorted it out using the whale optimization algorithm by way of finding the $n-1$ optimal n-level threshold. This enabled an effective optimization of operational parameters resulting in a sensitivity of 88.9% and specificity of 98.7%.

Christodoulidis et al. [35] noticed certain inadequacies in multiscale line detection while handling the smallest vessels, but combining it with multiscale tensor voting framework could circumvent this problem. Fragmented vessels could be connected in this approach, thus enabling detection of whole vessels irrespective of the background noise. Pandey et al. [36] used local phase-preserving denoising technique. A log-Gabor wavelet filter functioned as part of it, where the amplitude information of noise got decomposed, keeping the phase information intact. Noise removal was performed with the help of maximum entropy thresholding. Hessian MS filter coupled with hysteresis thresholding was another proposal by Rodrigues and Marengoni [37]. Alternatively, the attempt by Zhang et al. [38], mainly consisted of second-order Gaussian derivative and eigen value analysis which could also address CVR and parallel vessel constraints.Traditional approaches for segmentation are listed in Table 1.

**Table 1:** Traditional approaches for automatic segmentation of retinal vessels

Approach in brief	Challenges addressed
Filter based	OL [17, 21, 23], OL & SM [22], OL & IA [15], OL, SM & IA [14, 16]
Multiscale based	OL [30, 31, 39], SM [37], OL & SM [36], OL & IA [33], SM, CVR & PV [38], OL,SM & CVR [35]
Morphology based	OL [24], SM [26, 27],
Vessel tracing and tracking based	OL [28]

Obstructive lesions (OL), small vessel (SM), central vessel reflex (CVR), parallel vessel (PV) and imaging artifacts (IA)

ML methods: As many as eighteen publications, covering the period of review, have been summarized here.

The challenge from artifacts such as haemorrhages, drusen and exudates, treated as OL was addressed in the following approaches. Waheed et al. [40] selected features based on shape and intensity to train an SVM classifier. True positive rate (TPR) of 84.02% and false positive rate (FPR) of 97.49% were recorded. Distinct from the above, multi-fractal and fourier fractal features were fed to the same classifier by Ding et al. [41]. Tang et al. [42] introduced a new feature named influence degree of average intensity to sort out the complexity arising from the same problem. Pixels belonging to vessels appeared darker and caused a drop in the average intensity in its neighborhood, while the non-vessel pixels caused an increase in the same. Each pixel thus gets evaluated in asserting its influence on average intensity of the local row where the pixel is located. Preceding this evaluation, Hessian matrix was used by the authors to identify the candidate regions. SVM was used as the classifier in the final step. Sensitivity and specificity obtained were 82% and 97% respectively. Barkana et al. [43] selected pixel intensity statistics features with a combination of Fuzzy systems, ANN and SVM classifiers. Sensitivity, specificity and accuracy obtained were 72.2%, 98.4% and 95.02% respectively. Zhu et al. [44] used a collection of features with divergence of vector field. The resulting feature vector was employed to train weak classifiers with the help of classification and regression tree (CART). The AdaBoost classifier was trained for segmentation task. Kaur and Mittal [45], suggested a combination of shape and intensity based features to train the neural network (NN) classifier. The robustness of the method was assessed using different datasets and the average sensitivity of 85.43% and specificity of 97.94% were recorded. Moment invariant feature, instead of shape, was used by Vega et al. [46] while training the NN classifier. Aslani and Sarnel [47] made use of RF classifier known for its speed and information fusion capability. It was trained with a hybrid feature vector consisting of Gabor filter response, intensity, vesselness measures and B-COSFIRE filter response. Local information with discriminating ability for vessel and non-vessel pixels were thus assessed. Shah et al. [48] used regional, intensity and Hessian features to train a linear minimum square error classifier endowed with fast training capability.

The problem of small vessel missing (SM) which could arise from poor contrast was successfully handled in the attempts discussed below. Annunziata and Trucco, [39] employed scale and curvature invariant ridge detector for thin structures (SCIRD-TS) filters, which comprised of a learnt sequence of parameterized Gaussian filters with curvilinear support. This was followed by an RF classifier. An AUC of 0.87 indicated its performance. Strisciuglio et al. [49] explored a subset of B-COSFIRE filters selective for vessels of different thickness. An automatic selection process determined the subset, which was used to train the SVM classifier. Sensitivity of 80% and specificity of 97% were achieved. Oliveira et al. [50] used Gabor wavelet filtered features which were clustered by fuzzy C-means classifier resulting in a TPR of 86.44% and FPR of 4.44%. Memari et al. [51] managed both SM problem as well as OL interference simultaneously by pre-processing using CLAHE, B-COSFIRE and Frangi filters. This was followed by computation of pixel-statistics, texture and Gabor-based features, from which suitable features were selected and processed using AdaBoost classifier. Sensitivity and specificity values obtained were 87% and 98.8% respectively. Jebaseeli et al. [52] proposed a tandem pulse coupled neural network (TPCNN) model capable of extracting tiny vessels at their cross boundaries, which did not show up clearly due to depigmentation. A pixel-wise classification was achieved by employing deep learning based SVM (DLBSVM). Sensitivity of 80.27% and specificity of 99.8% were obtained. Parameters had to be set each time in this method. Later, they modified TPCNN [53] incorporating particle swarm optimization (PSO). This could assign values for its multiple parameters, thus adjusting the decay speeds of the thresholds according to each and every situation. Consequently, sensitivity and specificity values obtained were 94.68% and 99.70% respectively.

Fusing of closely parallel vessels is another problem affecting segmentation. Panda et al. [54] countered this problem with the help of k-means clustering and SVM classifier. Diabetic/hypertensive retinopathy-induced obscuring lesions interfering with segmentation efficacy was also sorted out by them, using edge distance seeded region growing technique coupled with SVM. Sensitivity and specificity values recorded were 84% and 95% respectively.

Attempts to overcome the obstacle of bifurcation/crossover point were made by several researchers. Kalaie and Gooya [55] classified the local intensity cross sections as junction or vessel points. A probabilistic graphical model was introduced by them for this purpose. Hyperparameters were assessed using a maximum likelihood solution based on Laplace approximation leading to a precision value of 88.67%. Srinidhi et al. [56] devised a visual attention guided unsupervised feature learning (UFL) method which mimicked the visual attention mechanism present in human visual system. As human beings do, this UFL also concentrates on the most important part of the image and neglects the remaining [57]. Non-uniform illumination and contrast variability were eliminated by suitable pre-processing. Image patches acquired from random locations were converted into differently sized glances. Smallest glance (G0), near the pixel of interest, provided the best spatial information, and the higher sized glances having lower resolution, provided contextual information. All these glances were then converted to the size of the smallest one using retinal transformation, followed by concatenation to afford an image patch embedded with both contextual and spatial information. Such patches carrying the most discriminating features, collected from several random locations were used to train a filter bank. The learned filter bank along with the set of labeled training images were maneuvered to acquire features which were subsequently fed to a RF classifier. Average values of 83% and 97% were obtained for sensitivity and specificity respectively. ML approaches of vessel segmentation are listed in Table 2.

**Table 2:** ML approaches for automatic segmentation of retinal vessels

Approach in brief		Challenges addressed
Classifiers	Features used	
SVM	Shape and intensity based features [40], Multi-fractal and Fourier fractal features [41], Edge distance seeded region growing technique, k-means clustering [54], Average intensity and Hessian feature [42]	OL
	Generalized matrix learning vector quantization [49]	SM
AdaBoost	Divergence of vector field and CART [44]	OL
	Pixel-statistics, texture and Gabor-based features [51]	OL and SM
NN	Shape and intensity based features [45]	OL
	Moment invariant pixel representation [46]	OL and SM
RF	Gabor filter responses, B-COSFIRE filter and Hessian matrix analysis [47]	OL
	SCIRD-TS filters [39]	SM
	Visual attention modelling, k-means filter learning [56]	BC, OL, SM, CVR, PV and IA
Fuzzy systems, ANN and SVM	Pixel intensity statistics features [43]	OL
Fuzzy C-means	Gabor wavelet filters features [50]	SM
Linear minimum squared error	Hessian and regional statistical features [48]	OL and CVR
Probabilistic graphical model	Intensity profile features [55]	BC
Deep learning based SVM	TPCNN features [52]	OL

Obstructive lesions (OL), small vessel (SM), Bifurcations/crossover points (BC), central vessel reflex (CVR), parallel vessel (PV) and imaging artifacts (IA)

#### Deep learning methods

Diabetic retinopathy (DR) manifests with damages in retinal blood vessels. Advances in image processing coupled with artificial intelligence and computer vision techniques have revolutionised the diagnostic precision of DR in ophthalmic practice. DL techniques have been successfully implemented in this area [58]. The retinal vessel map provided characteristic features such as vessel diameter, branch angles and branch lengths which helped the doctors to arrive at the pathology and initiate faster corrective measures [59]. Manual segmentation of the retinal vessels was a good old practice which, however ended up as expensive and time consuming process [60]. Lower contrast between vessels and backgrounds, uneven illuminations, variation in vessel width and shape introduced inconsistency in the results. DL methods emerged as a feasible solution, under these circumstances, with automatic feature extraction ability, thus minimising human interference.

In view of the exhaustive and pragmatic recent review of DL methods for vessel segmentation by Chen et al. [59], considering 89 DL models, spread over 5 years from 2016-2021, a detailed survey of methods is not included here. However, a brief summary is given for the sake of completion.

Prominent DL methods attempted for segmentation task were CNN, fully convolutional network (FCN), U-net and generative adversarial network (GAN). Substantial improvement in techniques were effected in each of the above, so as to attain better results. The basic CNN models could achieve only 94% accuracy [59]. Also, CNN lacks feature representation ability as it consists of simple convolutional layers. This permits only basic structure segmentation. Therefore, majority of the vessel boundaries and thin vessels got misclassified resulting in its less frequent usage [59].

FCN, an advancement over traditional CNN, incorporating more number of convolutional layers was capable of predicting each pixel in an image. Thus it was more suitable and fast for segmentation purposes with slightly better accuracy. However, it suffered from a drawback of blurry and smooth edges of segments [59]. These were sorted out with the help of U-net [61]. The commonly used and popular U-net based models could perform better than FCN which offered around 96% accuracy [59]. The U-net architecture is equipped with an encoder which extracts features. This is connected to a decoder which reconstructs the images. Both are connected by skip connections, the whole arrangement finally taking a U-shape. Hence the name U-net. The left-hand side of U-net represents contraction path (encoder) where the size of the image gradually reduces, while the depth gradually increases. Here, ‘what’ information gained momentum as it descends, while ‘where’ information is lost. Right-hand side represents the decoder, where the size of the image gradually increases as it ascends and the depth gradually decreases. Decoder recovers ‘where’ information by applying up-sampling. Skip connections join the encoder and decoder at every point and ensures better precision of the locations. In short, ‘what’ information from the encoder joins the ‘where’ information of the decoder at each skip connection point. It was this architecture which led to good performance with U-net. However, not contented with this performance, several other modifications, as many as 32, were effected on the basic U-net architecture by different researchers [59]. Among these, in a recent publication of Li et al. [62], employing attention mechanism and selective kernel units (SK units) on the basic U-net, 81.45% sensitivity and 97.69% accuracy was reported. Attention mechanism indicated which extracted features were to be focused on in a particular context, neglecting irrelevant information. It helped locate the region of interest and strengthen feature representation. A combination of self-attention and soft-attention modules (through SK units) was added in the basic U-net. Self-attention mechanism projected the correlation between each position and integrated local features with the global contextual information. Soft attention using global information helped highlight important features, at the same time suppressing noise generated features. Overall, the impression gained is that the attention mechanism used by Li et al. could extract more details compared to the baseline U-net model and attribute high scores for retinal vessel pixels.

In a recent study [63], the authors opined that the efficiency and precision of segmentation do not attain the expected levels when applied to the vascular ends and thin retinal vessels. Therefore, a different approach was adopted. Annotations were separately done for the original, thick and thin vessels which were then used to train the U-net model to obtain probability map for each of them. A pixel-wise classification on prediction probability maps was carried out. When the probability at any pixel point in any of the probability maps exceeded or became equal to the threshold value of 0.5, that pixel was considered as belonging to a vessel. This approach could provide an accuracy of 96.85%.

A recently introduced unsupervised learning model known as GAN consists of generator and discriminator models. Generator was meant to synthesize images (fake labels) while the discriminator, as the name suggests, was meant to sort out manually annotated vessel maps (real label) from the machine synthesized images [64]. GAN provided about 96% accuracy in segmentation matching that of U-net [59]. An architecture known as M-GAN employed by Park et al. [64] resulted in an accuracy of 97.06%. M-GAN derived the name from the two stacked deep FCNs with a multi-kernel pooling (MKP) block in between, giving an ‘M’ shape. Scale variance due to thick and thin vessels was attenuated by MKP. To achieve robust segmentation, residual blocks were made a part of the M-generator. M-discriminator used deeper networks along with residual blocks which reduced the associated vanishing gradient problem and at the same time imparted efficient training of the adversarial model. As part of training the discriminator, the machine generated and the original fundus images were linked so as to be considered as fake label. Similarly, the ground truth mask image was linked to the original image and considered as the real label. The learning was said to be complete when the machine generated map was judged as real by the discriminator.

Comparison of methods indicated that other than CNN, all other models performed almost equally ($\approx$ 96%) [59]. It may be noted that, U-net, with its symmetrical encoder-decoder structure and skip connections, integrated low and high level feature maps. Thus, local and global information could be obtained which led to improved segmentation. Finally, though not better accuracy-wise, unsupervised GAN showed an edge over even U-net, in its ability not only to synthesize several similar images, but also to label them [65]. This made available large number of suitable images to train DL models. Adversarial learning process could be considered as another contribution of GAN.

### Arteriovenous classification

**Fig. 2:**
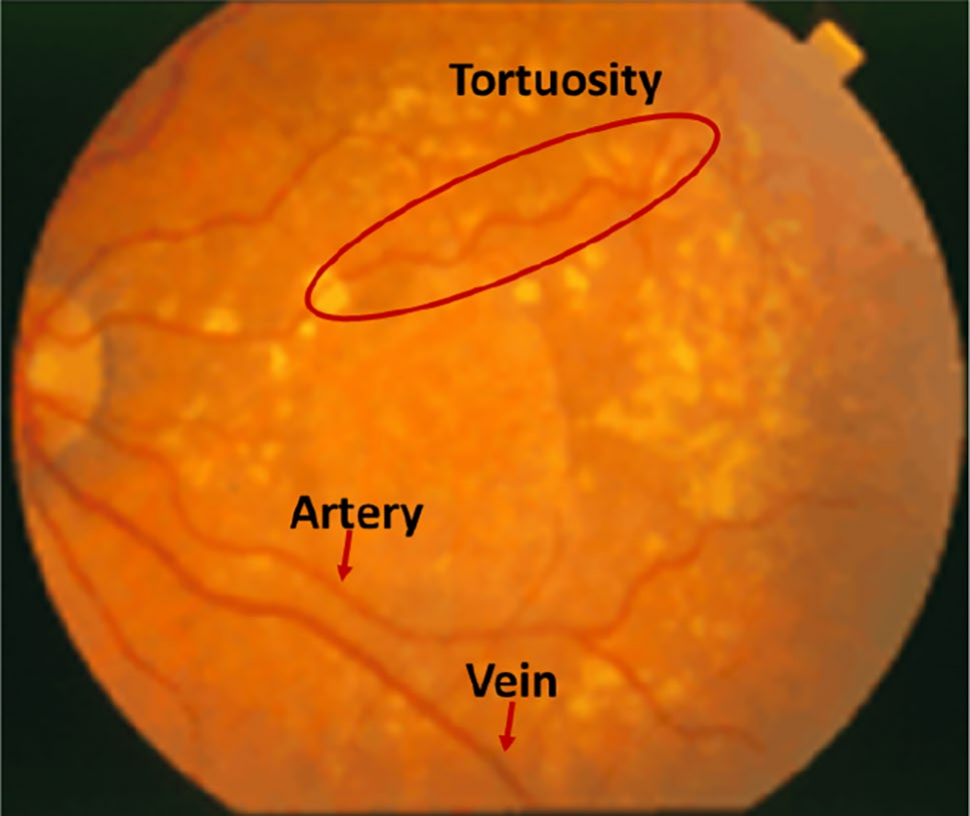
Image indicating different thickness and color for arteries and veins from STARE database

Vascular abnormalities such as arterial narrowing and venous vessel bleeding could show up different diagnostic features at different stages of retinal diseases [66]. Also, arteries and veins are considered separately in the measurement of oxygen content of blood. Morphologically, veins are straighter and wider than arteries, but colorwise they are darker as shown in Fig. 2. In the following sections "Traditional Methods" and "DL Methods", details of different traditional and DL methods respectively, are presented.

#### Traditional methods

Accurate and efficient computer aided methods of classification are required to diagnose and treat various ailments especially eye diseases using color fundus images [67]. In the supervised ML category, Xu et al. [68], addressing the OL problem, made use of the vessel profile, color, texture and Gabor-based orientation features as input to the KNN classifier, thus achieving an accuracy of 92.3%. Zhu et al. [69] proposed a method to extract five types of features related to local intensity, morphology, phase congruency, Hessian and divergence of vector fields. Divergence measures the rate of change in the strength of the vector field at a given point. A positive value indicated the presence of blood vessels. The above set of features were fed to an extreme learning machine (ELM) classifier, which functioned as a simple feedforward NN. It enabled faster training as it had a single layer of hidden nodes. Lesions and background got suppressed in this procedure, thus providing an accuracy, sensitivity and specificity amounting to 96%, 71% and 99% respectively. Akbar et al. [70] used SVM classifier providing color and statistical features, achieving an accuracy of 95.6%, sensitivity and specificity values of 98% each to spin out the same problem. Retinal vessel characterization attempted in the method of Yin et al. [71] mainly depended upon statistical measures of histogram, response of different filters and local gradient aspects. Best feature selection from among the several features available was carried out using Pearson correlation coefficient technique and Relief-F method. Three classifiers namely KNN, SVM and Naïve Bayes were primarily used to carry out the classification task. Sensitivity of 95.52%, specificity of 92.34% and accuracy of 93.90% were achieved. Arterial narrowing, a marker for hypertensive retinopathy in its initial stages could be quantitatively assessed using the proposed method.

In order to subdue the SM problem, Yan et al. [67] used a combination of shifted filter responses which enabled discrimination of vessels from non-vessels. Morphology, topology and context based features were then selected and applied to a JointBoost classifier. Accuracy of classification with respect to artery and vein was 94.5% and 91.1% respectively.

To transcend the BC issues, Vijaykumar et al. [72] proposed RF classifier so as to find the most suitable features. These were furnished to train the SVM classifier. Sensitivity, specificity and accuracy obtained were 90.87%, 94.82% and 92.4% respectively. To get over the same hurdles, Pellegrini et al. [73] proposed Bayes classifiers and graph cut on ultra-wide field of view-scanning laser ophthalmoscope (UWFoV-SLO) images. Srinidhi et al. [74] presented their work in the following sequence: identification of vessel key points, graph representation of the vascular network, vessel subtree extraction and subtree A/V labeling. Key points of the vessels were identified, following which topological and spatial connectivity were featured in a graph representation. Vessel crossing and branching points possessed certain unique features. Based on these, the vessel tree was divided into subtrees comprising of arteries and veins. In order to label the subtrees as artery or vein, a RF classifier was trained with two additional features namely line detector response and histogram of oriented gradients. This resulted in 94.7%, 96.6% and 92.9% of accuracy, sensitivity and specificity respectively.

In the unsupervised approach, a recently published method [75] claimed many advantages over the existing methods in view of the inherent challenges associated with A/V classification. It was a common observation, that in case of narrow vasculature, difficulty arose in discriminating arteries from veins, even if good segmentation was achieved. The complexity increased when bifurcation problems also existed. Yet another problem was that of crossover. Such intersections led to ambiguity in their appearance, contrast and geometry. This challenge has been addressed by the method proposed by Zhao et al. [75], the concept of which centered on dominant set clustering for vessel topology reconstruction. This method comprised of segmentation, skeletonization, overlaying significant nodes on the skeletonised image and reconstruction of the vascular network based on edge weight assessment in the feature space. The resulting vascular network was classified into arteries/veins taking into consideration their intensity and morphology. Sensitivity, specificity and accuracy obtained were 94.2%, 92.7% and 93.5% respectively. ML approaches of vessel classification are listed in Table 3.

**Table 3:** ML approaches for vessel classification

Approach in brief		Challenges addressed
Classifiers	Features used	
KNN	Color, texture and Gabor-based features	OL [68]
ELM	Phase congruency, Hessian and divergence of vector fields features	OL [69]
SVM	Color and statistical features	OL [70]
KNN, SVM and Naive Bayes	Statistical measures of histogram, different filters response and local gradient aspects	OL [71]
JointBoost	Morphology, topology and context based features	SM [67]
RF and SVM	Intensity and morphological features	BC [72]
Bayes	Graph cut approach	BC [73]
RF	Descriptors for vessel points, vascular graph representation, line detector response and histogram of oriented gradients	BC [74]
Dominant set clustering for topology reconstruction	Intensity and morphology based features	BC [75]

Obstructive lesions (OL), small vessel (SM), Bifurcations/crossover points (BC)

#### DL methods

Retinal artery-venous ratio (AVR) is known to give a good indication of the cardiovascular risk and its severity. Accurate A/V classification, therefore, is of paramount importance. In the method proposed by Girard et al. [76], CNN and graph propagation strategies were combined. Though the basic CNN provided a pixel-wise classification expressing the likelihood score for an artery or vein, it did not focus on structure of the vascular tree. The above scores were aggregated into a vessel branch score and transmitted into a vascular network using a graph representation. The CNN output was efficiently propagated and refined through the minimum spanning tree of the graph. This approach could achieve an accuracy of 93%. In a revised proposal, they attempted joint segmentation and classification using a scalable encoding-decoding CNN model, keeping up the same efficiency as those of state-of-the-art methods, but speeding up the inference considerably [77]. Xu et al. [78] in their proposal suggested a FCN feature representation in U-net, with an additional domain-specific loss function. Hemelings et al. [79] used FCN feature representation in U-net, to which RGB input was given, for increasing the information per pixel. They created a suitable A/V ground truth. Provision for ternary labeling as background, arteries and veins was still another contribution which helped them to achieve 97% accuracy.

**Fig. 3:**
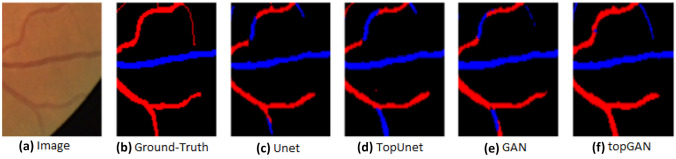
A/V classification results on DRIVE dataset [81]

Ma et al. [80] aimed at vessel segmentation and A/V classification simultaneously. For this purpose, U-net architecture with pre-trained ResNet as the encoder was used. Two parallel branches, one for segmentation and the other for A/V classification were arranged at the network terminus to provide multi-task (MT) output. Common features of both artery and veins were captured by one of these branches. The discriminating features between the two were handled by the other branch. The emergent maps were effectively combined to process the result as A/V classification. This was performed by the spatial activation mechanism which considered higher weights for capillary vessel pixels and lower weights to the thick vessels. In addition, the information gained from retinal preprocessing and vessel enhancement techniques were also integrated with the help of a multi-inputs (MIs) module. Thus, full vessel segmentation as well as A/V classification were carried out simultaneously utilizing all the three components MT, MIs and spatial activation mechanism. Classification performance was indicated by an accuracy of 94.5%, sensitivity of 93.4% and specificity of 95.5%. Yang et al. [81] attempted to rectify certain drawbacks in the existing intensity based and graph based A/V classification methods. In the intensity based method, topological information was lacking. In the graph-based methods, as vascular structural characteristics were taken into account, there was a need for careful evaluation of BC, failing which misclassification of the sub-trees of retinal vessels could occur. Further, non-connectivity could also creep in as a drawback in segmentation even in the DL methods. To resolve these issues, topological structure-constrained GAN (topGAN) was suggested. Firstly, it generated an A/V segmentation map. A topological structure loss function was then made use of, which could clearly describe the complex vascular structure. The combination of topological factor with GAN was thus able to improve the connectivity of A/V classification, in comparison with other methods specified in Fig. 3. Thus, the chances of assigning artery as vein mistakenly are reduced. Accuracy, sensitivity and specificity reported were 93.9%, 90.7% and 92.6% respectively.

A recent publication by Hu et al. [82] summarized the efforts to alleviate the problems that were encountered with SM detection. In retinal fundus images, the blood vessels are confined to 15% of the image, with arteries and veins contributing only to 7.5% each, which makes the task all the more challenging. There was even a possibility of recognizing one half of the blood vessel as artery and the other half as vein. Generalization capability was found to be poor when tested on images with different resolutions and scales. A CNN named vessel-constraint (VC) network was devised for this purpose. It consisted of VC module which suppressed background features and at the same time enhanced edge features of blood vessels. The other one was multiscale feature module providing blood vessel information in different scales. Accuracy of 95.54%, sensitivity of 93.6% and specificity of 97.48% reflected the performance efficiency. DL approaches of vessel classification are listed in Table 4.

**Table 4:** DL approaches for vessel classification

Approach in brief	Challenges addressed
CNN with graph propagation strategies [76]	OL and SM
Scalable encoding-decoding CNN model [77]	OL and SM
FCN feature representation using U-net [79]	SM
Multi-task, multi-input and spatial activation on U-Net for simultaneous segmentation & classification [80]	PV
Topological structure-constrained GAN [81]	OL
CNN with vessel-constraint & multiscale feature module [82]	SM

## Corneal nerve images

**Fig. 4:**
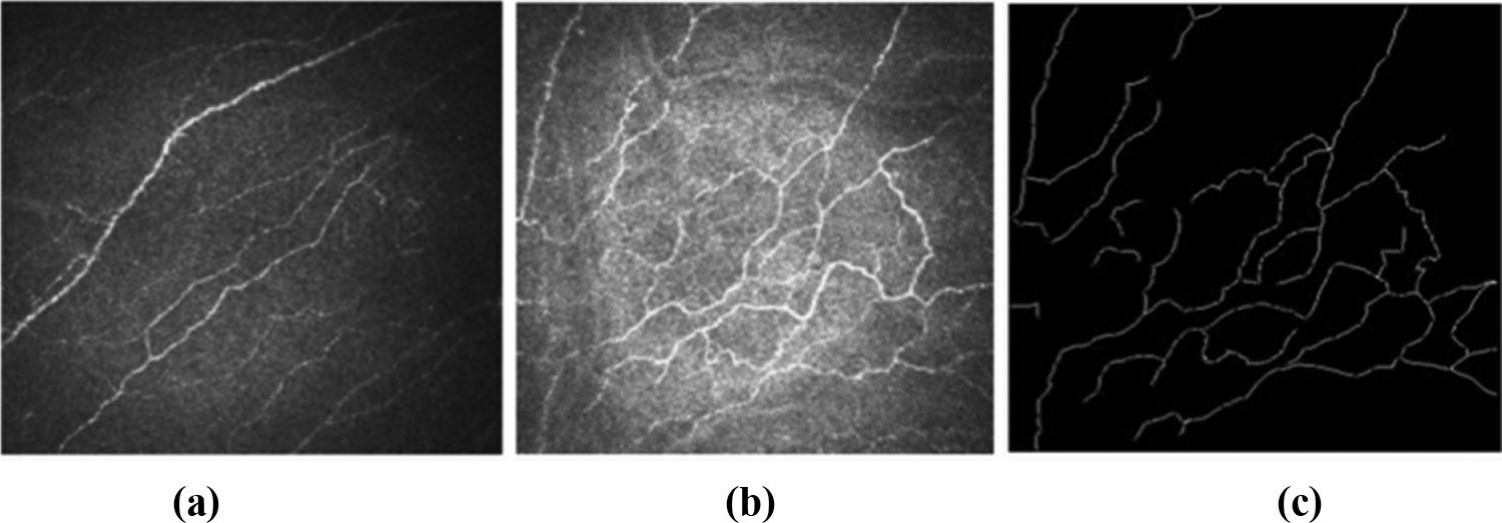
Image of corneal nerve fibers from CORN dataset **a** Almost straight **b** Highly tortuous **c** Labelled image of (**b**)

Quantitative analysis of corneal nerves have been reviewed adequately in three review papers published in 2018 [83], 2019 [84] and 2021 [85]. Petropoulos et al. [86] suggested corneal confocal microscopy (CCM) to be the final step to arrive at conclusions related to peripheral and central neuro-degenerative conditions. In-vivo confocal microscopy (IVCM) images as applied to cornea are also known as CCM images. In IVCM images of the cornea, not only do the nerves follow a particular direction, but also appear as conspicuous bright lines in a noisy background [87]. This technique permitted a non-invasive examination of the different layers of the cornea aimed at assessing the corneal health status. Visualization of the narrow and elongated nerve structure at the sub-basal layer revealed pertinent clinical information regarding ageing-induced changes, effects of surgical interventions, dry eye syndrome and keratoconus. Keratoconus, is a condition wherein the dome shaped tissue covering cornea thins and bulges outward into a cone shape. Relation between nerve tortuosity and severity of neuropathy resulting from long-term diabetic condition could also be studied. Images of nerve fibres with different levels of tortuosity can be seen in Fig. 4.

Automated procedures, free from the implicit defects of manual tracing such as subjectivity and delayed retrieval of clinical information are much desirable in a busy clinical set up. Length, density and tortuosity of the nerves along with their path and layout could give critical information helpful in accurate diagnosis [88]. Automated segmentation of nerves from the images procured from CCM to capture the features, and classification of images into healthy or diseased types, were steps considered inevitable for a successful diagnosis. In the following sections "Traditional Methods" and "Deep Learning Methods", details of different traditional and DL methods respectively, are presented.

### Traditional methods

Tavakoli et al. [89] made attempts to assign age-adjusted normative values of corneal nerve fiber parameters essential for clinical translation and wider use of CCM. The images were processed using CCMetrics software designed by Dabbah et al. [87] which is briefly summarized here below.

When a fixed wavelength for Gabor filter component was used depending on the nerve thickness, performance was not satisfactory. It was, therefore, improved by Dabbah et al. [87] by suitable alteration of the angular orientation and spatial scale of the kernel filters. With the help of their multiscale dual model, they enhanced the nerve fiber images using 2D Gabor wavelet filter along with an additional Gaussian filter. Response from these filters was fed to an artificial neural network (ANN) for classification. Sensitivity and specificity values obtained were 84.78% and 84.67% respectively. Decrease in equal error rate (EER), indicating good accuracy in performance was achieved. It was accepted as a standard software application known as ACCMetrics and put into use in 2015 by the University of Manchester, UK [89].

In the detection of a variety of curvilinear structures, the multiscale method was found to be useful. Annunziata et al. [90] designed a method for segmentation and tortuosity estimation. The segmentation module used SCIRD and a context model to analyse tortuous and fragmented structures. Context model is a group of filters which enables identification of the more discriminative features such as curvature and the points where the sign of curvature changes. The tortuosity estimation module computed the tortuosity measures in a scale-space representation. Time required to analyse an image was 30s. Accuracy of segmentation varied from 72% to 78%; sensitivity value varied from 52% to 58% and specificity value varied from 74% to 84%. The authors opined that multiscale approach outweighs the single-scale approaches specifically in tortuosity estimation. A morphology based technique proposed by Al-Fahdawi et al. [91] consisted of two steps. The first one being background reduction using a combination of coherence and Gaussian filters. The second step consisted of morphological approaches to eliminate unwanted segments. Canny edge detection was used to detect nerve fiber in the image. Gaps in the fibers were filled by applying a suitable algorithm. It consisted of conversion of the image into a skeleton followed by marking a circle at the endpoints of each segment. Disconnected end points were joined with the help of a straight line depending upon the distance between them. When thinning operation was applied on the image, the circles shrunk into a line. Clinically important features such as nerve tortuosity, length, density and thickness were measured from the segmented image. Fairly shorter time of 7s was claimed for processing a single image, but the setback was that no comparison of conventional metrics had been made with those of other methods in the literature. In another method devised by Hosseinaee et al. [92] edge enhancing diffusion technique was used, followed by Hessian-based multiscale filtering to highlight nerve structures. Adaptive threshold helped detection of corneal nerve edges. Morphological operations could remove noisy background and also connect disjoint nerves. Processing time was 18-20s. However, good tracing quality was claimed.

Silva et al. [93] extracted 61 texture based features which were reduced to six with the help of principal component analysis (PCA), in order to avoid redundant features. SVM was then applied to classify images into normal or mild/moderate neuropathic types. An accuracy of 73.5% in identifying images as neuropathy/non-neuropathic classes was achieved. Accuracy achieved for mild or moderate classification was 79.3%. However, the dataset of images being procured from only 20 subjects lacked diversity. Another morphology based enhancement coupled with SVM classifier was the basis of the method proposed by Guimaraes et al. [1]. Top-hat aided enhancement was followed by log-Gabor filtering which helped pattern recognition. For the purpose of nerve segmentation, hysteresis thresholding was applied which projected true and false nerve segments. Output from a series of filters including log-Gabor filters were differentiated using SVM. A sensitivity of 88% was achieved with 8% false detection rate (FDR), having a processing time as low as 0.61s. Salahuddin and Qidwai [94] tried to select a good classifier from among the different known algorithms namely SVM, Naïve Bayes, linear discriminant analysis (LDA), decision trees and KNN. Images were first reduced in size to one-fourth of the original using discrete wavelet transform and then subjected to Gaussian coherence filtering so as to avoid background noise and enhance the linear structures. Binarization was effected followed by morphological operations to ward off residual noise and connect the broken segments. Three features namely area of the nerves, entropy and nerve fiber length were extracted and fed to the above mentioned classifiers. KNN was found to be the best classifier with an accuracy of 92%. In these segmentation techniques, handcrafted features play a crucial role, whereas, in DL techniques, there is a definite advantage of not needing any separate segmentation step or manual feature extraction.

### Deep learning methods

CNN has frequently been exploited to assign images into specific classes. However, in biomedical image processing, there is a dire need to assign a label to each pixel instead of an image as a whole. U-net [61] has been one of the popular architectures that was used for this task. Requirement of limited labeled data was an advantage with this architecture [95]. U-net and U-net like models have thus become acclaimed models in segmenting variety of biomedical images including those of neurons and vascular boundary [96]. Colonna et al. [97] proposed a method for segmentation followed by classification of CCM nerve images obtained from healthy and diabetic patients. They trained a U-net based CNN with 8909 images, 30% of which was used for validation. Only 30 images were used for testing. Sensitivity of 97% and FDR of 18% were recorded. Williams et al. [98] used an ensemble of five U-net networks arranged in parallel, the final prediction being derived from the majority vote from all the five U-net models. Sensitivity and specificity reported were 68% and 87% respectively. Zhang et al. [99] introduced attention gate (AG) module, to suppress irrelevant features and to emphasize the more relevant ones. It resulted in a sensitivity of 86.32% and specificity of 99.78%. Mou et al. [100] in a recent attempt to segment corneal nerves proposed a CS2-Net approach consisting of three modules: encoder module, channel and spatial attention module and decoder module. The encoder extracted the input data features. The extracted features are fed to two parallel attention blocks. The spatial attention block enabled the module to identify the long-range dependency of the features. Thus, similar features got related, irrespective of their distance. The channel attention block helped increase the contrasting ability of different features in different channels which upgraded the discrimination ability of the model. A comparative evaluation of their proposal with methods employed by other researchers [61, 101–104] for nerve fiber tracing revealed a sensitivity of 84% and FDR of 25%. Suppression of background interference was found to be good in CS2-Net when compared to U-net. The CS2-Net approach proposed by Mou et al. was validated across six imaging modalities of curvilinear structures such as retinal color fundus image, retinal optical coherence tomography angiogram, CCM image, Optical coherence tomography (OCT) and Brain magnetic resonance angiography. Wei et al. [105] employed a CNN architecture with ResNet34 as the encoder to enable feature extraction and a decoder, in order to analyse the feature map for segmentation. Skip connections were introduced between the two to upgrade the information flow from low-level to high-level features. A new loss function was also incorporated combining dice coefficient loss, mean square error and regularization loss. These modifications resulted in a sensitivity of 96% and specificity of 75%.

**Fig. 5:**
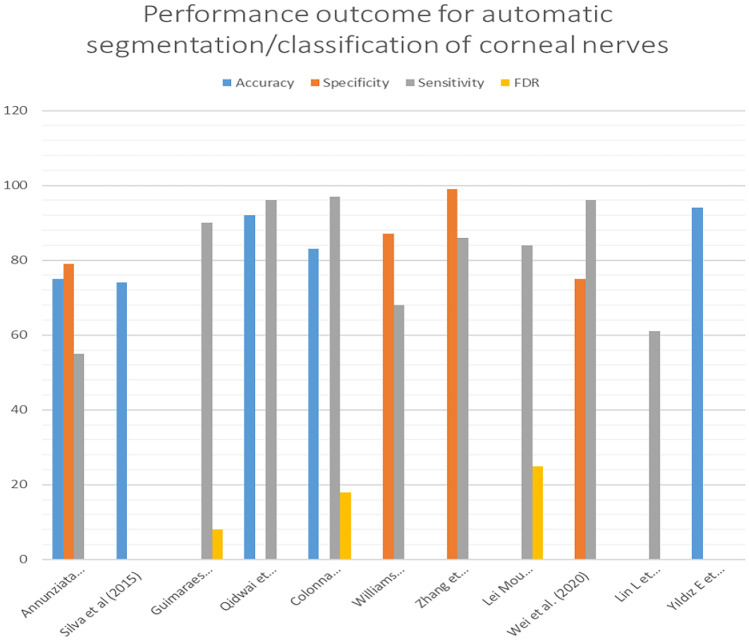
Performance outcome of corneal nerve segmentation/classification methods

In a recent study by Lin et al. [106], though the basic U-net model has been employed for segmentation in the entire pipeline proposed, emphasis has been given to image quality enhancement integrated with contrastive learning approach, raising the dice score value from 0.76 to 0.82. Multi-layer and patchwise contrastive learning based GAN has been put to use to take care of multiscale local features. Inadequate annotations which would have resulted while handling very narrow nerve structures, unequal illumination and contrast differences were suggested to be hindrances in the segmentation pathway. The proposed method was claimed to overcome these hindrances. In another recent article, Yıldız et al. [107] compared the performance efficiencies of U-net with conditional GAN (cGAN). In the latter, it was necessary to assign class labels to the input, so that targeted image generation of the given type could be achieved, whereas in original GAN, the input lacked any labels [108, 109]. A neck and neck comparison of U-net and GAN has been projected by the authors wherein, cGAN had an edge over U-net in accuracy. For example, the accuracy of GAN network was not affected by adding noisy input, while that of U-net showed a decreasing tendency [107]. U-net had a tendency to produce more false positive results in the presence of artifacts such as non-uniform illumination, device-related noise and distractors like dendritic cells/damaged nerves. A brief summary of the methods and challenges addressed is presented in Table 5. Performance outcome for the above methods is given in Fig. 5.

**Table 5:** Method outlines and challenges addressed for automatic segmentation/classification of corneal nerves

Study	Approach (Dataset: No. of images)	Challenges
Annunziata et al. [90]	Segmentation: SCIRD along with multi range context filters (IVCM:140)	a) Poorly contrasted, fragmented and highly tortuous fibres. b) Dendritic cells mimicking nerve fibres with similar appearance
Al-Fahdawi et al. [91]	Segmentation: Morphological operations, Canny edge detectors (CCM: 498)	Discontinuity in nerves and noise
Hosseinaee et al. [92]	Enhancement: Hessian based filtering; segmentation: Thresholding (In-vivo OCT corneal nerve images: No. unspecified)	Eye-motion-induced blurring of images and discontinuity in nerves
Silva et al. [93]	Classification: Texture based features, SVM classifier (CCM: 631 images)	Complex, low quality diabetic peripheral neuropathy images
Guimaraes et al. [1]	Nerve tracing: Hysteresis thresholding, SVM classifier (CCM: 246)	a) Reduction in processing time b) Illumination artefacts, bright elongated structures like cells resembling corneal nerves
Salahuddin and Qidwai [94]	Segmentation: Gaussian coherence filtering, thresholding; classification: KNN (CCM: 297)	a) Noisy background b) Linking of fragmented segments
Colonna et al. [97]	U-net based CNN for segmentation of corneal nerves and classification of images into healthy and pathological (CCM: 8909)	a) Large execution time b) Need for manual refinement c) Class imbalance problem.
Williams et al. [98]	Segmentation using ensemble of U-nets (CCM: 3835)	Accuracy enhancement
Zhang et al. [99]	Segmentation using U-net incorporating attention gating (AG) modules CNM (corneal nerve fiber images of Maastricht: 142)	a) Poor nerve fiber visibility b) Background artifacts
Mou et al. [100]	Segmentation: Encoder-decoder architecture with spatial and channel attention mechanisms (CORN-1: 1698)	a) Methodology acceptance over widely varying medical imaging modalities b) Adaptability to 3D images
Wei et al. [105]	Segmentation with Encoder (ResNet 34)-decoder architecture (IVCM: 671)	Speed enhancement compared to manual annotation
Lin L et al. [106]	Segmentation: U-net with cGAN (CORN:1578, CCM26:26)	Inaccurate annotations, non-uniform illumination and contrast variations
Yıldız et al. [107]	Segmentation: cGAN (IVCM: 505)	Accuracy improvement in noisy images

## Filamentous fungi

Segmentation/ classification attempts of dermatophytes/non-dermatophytes and keratitis causing fungi are included in sections "Dermatophyes/Non-dermatophytes" and "Fungal Keratitis" respectively.

### Dermatophyes/ Non-dermatophytes

The main focus in the study of filamentous fungi has been on industrially important specimens rather than on medically significant species till 2015. Several publications appeared in the last three decades dealing with identification of filamentous fungi from their cultures. Many of these methods were mainly aimed at quantification of fungal growth in fermentation procedures. Initial efforts for automation started in early 1990s. Reiehl et al. [110] developed a differential staining method of using Acridine Orange for better visualization of fungal septa which indicate cross walls in the filaments. These were seen as dark spaces in an orange stained cytoplasm. The interseptal distance between branches was computed from the resultant binary image. Using a digital image processing system, accurate and reproducible analysis of positional information on septa and branches was obtained. Visibility of septa was not optimum with light microscopy, but could be improved by using a suitable stain in fluorescence microscopy. This spurred interest in automation studies of filamentous fungal images, but were confined to only leaf and fruit fungus, besides industrial fermentation related species. Isolated efforts for automation in medically important fungal detection could be seen in the works of Mader et al. [111], Qiu et al. [112] and Wu et al. [113]. The following two sections "Traditional Methods" and "Deep Learning Methods" describe traditional and DL attempts, respectively.

#### Traditional methods

Mader et al, [111] detected dermatophytes, a pathogenic filamentous fungi, from digital fluorescence microscopic images of skin samples in the presence of challenging artifacts such as fibres from clothes, air inclusions and other miscellaneous objects such as dirt. Segmentation was performed using Canny algorithm. Binarization was carried out and the discontinuous objects were connected by morphological processes. The singled out regions of interest were extracted using a region growing algorithm and subjected to morphological and statistical feature analysis. The artifacts could be distinguished from the filaments of fungi, known as hyphae, due to variation in intensity and shape which appeared differently from those of the hyphae. Sensitivity and specificity obtained on clinical samples were 83% and 79% respectively. Speedy diagnosis was thus possible.

#### Deep learning methods

Other than the traditional approach by Mader et al. in 2015, a DL method for filamentous fungal detection using ResNet50 model was attempted by Gao et al. in 2021 [114]. Sensitivity and specificity recorded were 97% and 98% respectively. These methods used fluorescent microscopic techniques to acquire the images. The recently published work on dermatophyte detection using direct microscopy with potassium hydroxide treatment, devoid of fluorescence staining was comparatively more cost effective [115]. It focused on object detection using YOLOv4. It is known to possess higher speed of processing and performance compared to its previous versions. Average precision and speed have been reported to be higher than the earlier versions, rendering it suitable for fungal detection in real-time clinical practice [115]. The authors employed images of 100x and 40x magnification for this study. Sensitivity and specificity achieved were 95.2% and 100% in the 100x data model, and 99% and 86.6% in the 40x data model respectively. Another study employed VGG16, Inception V3, and ResNet50 for classification of fungi. Two approaches namely training from scratch and transfer learning were tried with all the three models. An accuracy of 73.2% was reported with Inception V3 model trained from scratch, and 85.04% for VGG16 using transfer learning approach [116]. However, the dataset they depended upon for training purposes consisted of images from non-dermatophytic moulds.

**Fig. 6:**
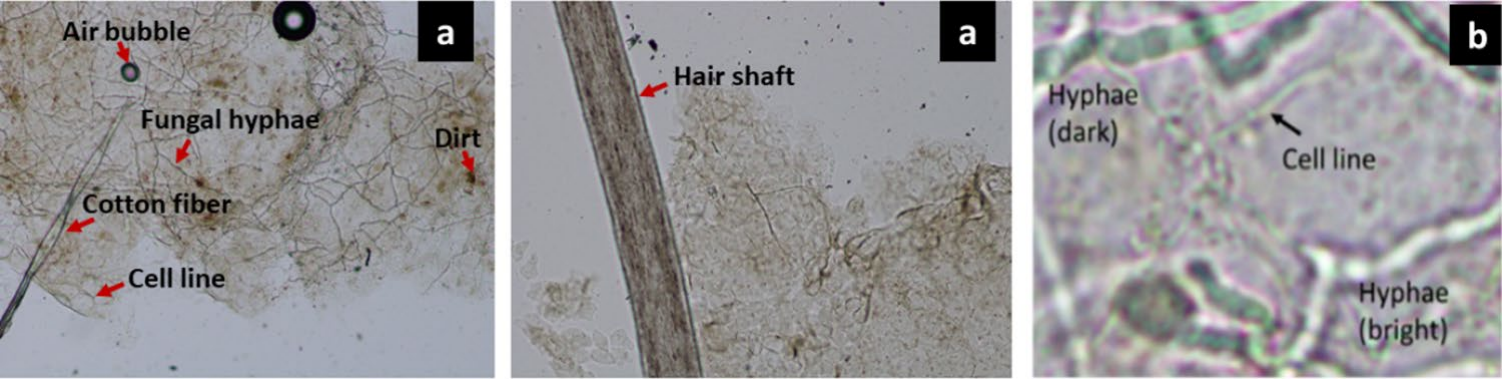
Examples of fungal microscopic images **a** 100x **b** 400x [117]

Examples of fungal microscopic images in different magnifications showing hyphal filaments and artifacts can be seen in Fig. 6.

### Fungal keratitis

Fungal keratitis, an infection of the cornea which can be caused by 70 different fungi is of utmost concern especially in its endemic areas for fear of causing blindness, if not diagnosed and treated properly [118]. It is reported that IVCM though widely used and highly sensitive, suffers from the problem of distinguishing hyphae from nerve fibres of the cornea. It calls for lot of expertise and experience to sort out the complexity due to structural similarity [112]. A brief description of methods focusing on traditional and DL efforts for diagnosis of fungal keratitis is given in the following two sections "Traditional Methods" and "Deep Learning Methods" respectively.

#### Traditional methods

Qui et al. [112], suggested the technique of texture analysis with the help of local binary patterns (LBP). SVM was used as a classifier. It enabled separation of images of healthy cornea from those of infected ones with an accuracy of 93.53%. Wu et al. [113], incorporated a new version of adaptive median binary pattern (AMBP) known as adaptive robust binary pattern (ARBP) for texture analysis. Instead of confining the threshold value calculation into a small region as in LBP and MBP, AMBP employed a larger adaptive window which captured richer texture resulting in optimal threshold value. Considering the higher brightness of nerves and hyphae compared to the background, average pixel value of the analysis window was treated as a parameter in ARBP. This resulted in a performance accuracy of 99.74%. Further, line segment detectors were used for hyphae detection.

#### Deep learning methods

**Fig. 7:**
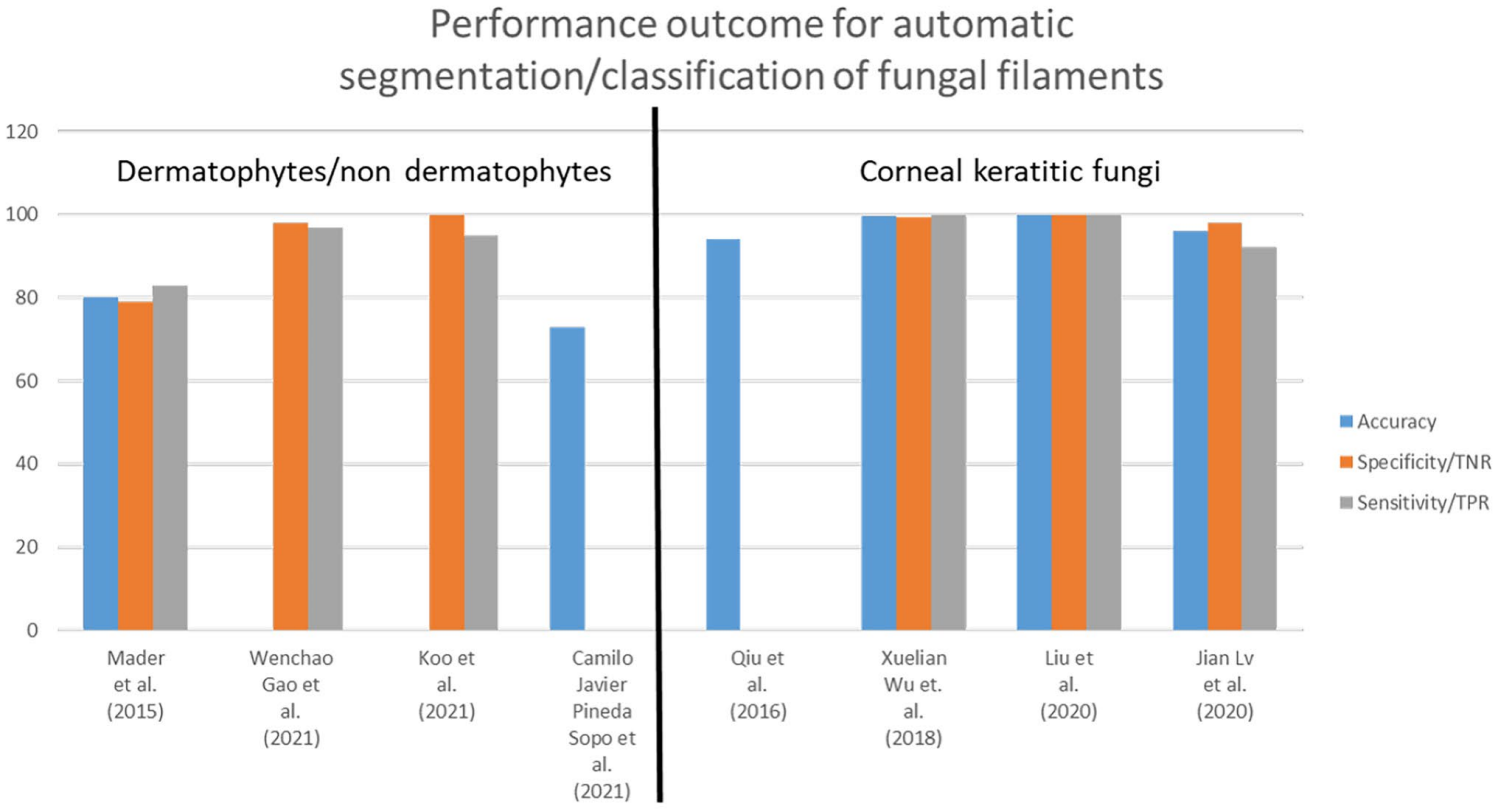
Performance outcome of fungal filament segmentation/classification methods

Lv et al. [119] employed ResNet for classification, achieving an accuracy of 96% with sensitivity and specificity of 91.86% and 98.34% respectively. Liu et al. [120] observed that the background in microscopic images of fungal keratitis was disordered and complicated with several unwanted lesions and spores. Detecting hyphae in the presence of these structures was difficult. Data augmentation by vertical and horizontal flipping helped to increase the number of images. Pre-processing using sub-area contrast stretching avoided information loss. A modified mean fusion method named histogram mean fusion (HMF) when adopted, resulted in enhanced key structures. The new dataset thus obtained was processed through AlexNet and VGGNet. A diagnostic efficacy of 99.95% was achieved by HMF-AlexNet combination. A 0.6% increase in accuracy was noticed compared to the AlexNet without HMF step. Summary of the methods is presented in Table 6. Performance outcome for the above methods is given in Fig. 7.

**Table 6:** Method outlines and challenges addressed for automatic segmentation/classification of fungal filaments (dermatophytes/non-dermatophytes and corneal keratitis)

Study	Approach (Dataset: No. of images)	Challenges
Dermatophytes/non-dermatophytes		
Mader et al. [111]	Segmentation: Canny edge detection, binarization. Classification: Morphological and statistical features (Stained dermatophyte images: 415)	Fibres from clothes, air inclusions, circular/irregular reflections and other miscellaneous objects such as dirt
Gao et al. [114]	Detection: ResNet50 (Stained filamentous fungi: 1175)	Reduction of detection time
Koo et al. [115]	Object detection: YOLOV4 (Unstained dermatophyte images: 3,707)	a) Presence of non-hyphae objects. b) Hyphae location in lower magnification.
Sopo et al. [116]	Classification: VGG16, Inception V3 and ResNet50 model (Different types of fungi-staining unspecified: Total-599)	a) Structural similarity among fungi types. b) Artifacts.
Corneal keratitic fungi (classification features/methods)		
Qiu et al. [112]	LBP and SVM (CCM: No. unspecified)	Complex and messy background, uneven brightness of images, threadlike appearance of hyphae and nerves.
Wu et. al. [113]	ARBP and SVM, hyphae detection using line detectors (CCM: 378)	a) Feature extraction of images with bright and small targets. b) Structural similarity of hyphae and nerves.
Liu et al. [120]	AlexNet with HMF algorithm (CCM:1213)	Cluttered background of images in infected samples, lesions and different types of fungal hyphae.
Lv et al. [119]	ResNet (IVCM: 2623)	a) Pathological cornea complicated by hyphae and inflammatory cells. b) Decreased No. and continuity of nerve fibres in diabetic cornea

## Discussion

In sections "Retinal Blood Vessels", "Corneal Nerves" and "Fungal Filaments", we have organized the discussions on methods dealing with retinal blood vessels, corneal nerves and fungal filaments, respectively.

### Retinal blood vessels

In view of the complications that could arise in eye diseases including loss of vision, the need for accurate methods to overcome challenges faced in image analysis cannot be overemphasized. There were many attempts in this regard as examples from traditional, ML and DL categories. Methods addressing important challenges have been presented in Tables 1-4. In both traditional and ML methods, performance accuracy ranged between 93% to 96%. Only a few publications crossed this performance level [14–16, 40, 44, 51–53, 55] and came upto 99% [52]. OL and SM challenges were given due weightage by most of the researchers as is evident from Tables 1-4. Discovering new trends and patterns which the humans cannot comprehend was the definite advantage of ML. ML was frequently explored where adaptation was a necessary component in complex images. OL, a frequently encountered impediment in A/V classification was solved considering intensity, morphology, phase congruency, Hessian, color and statistical features with the help of classifiers namely, ELM, KNN and SVM. Srinidhi et al. [56] addressed most of the challenges in segmentation with the help of visual attention mechanism. This method depended upon features learnt directly from the raw data instead of handcrafted features based on domain knowledge. It also adapted easily to various datasets, where availability of sufficient training sets and labeled annotations were limited.

A glance at the various DL models [59] and their modifications indicated that U-net with residual block [121], dilated convolution [122] and attention mechanisms [123] improved the detection capabilities considerably. In addition, several other GANs including M-GAN were found to be useful to enhance performance. Accuracy was found to be in the range of 96% [59] to 97% [64]. More details are discussed in an exhaustive review published by Chen et al. [59] on DL methods.

A few observations related to DL methods are mentioned below.

- Though large number of fundus images can be obtained, labeled data availability was found lacking, as the involvement of clinicians was a requirement.
- Many image samples had the problem of noise, non-uniform illumination, poor contrast for thin vessels and distractions such as pathological regions, fovea and macula which interfered with learning of features by the selected models.
- Vessel pixels being low in count compared with the larger percentage of background pixels, a class imbalance problem was faced, affecting the training efficiency.
- Thin vessels faced the misclassification problem more than the thicker ones. Most of the publications reported an average A/V classification accuracy less than 95%, considering the performance in different databases. However, a few other reports [69, 70, 79, 82] could achieve a performance beyond this level.

Despite all the efforts made by different researchers hitherto, there exists enough scope to enrich the DL performances especially when confronted with challenges such as vessels in cross connections, reconnection of fractured vessels and achieving robustness across different databases.

### Corneal nerves

A conclusion based only on the results of sensitivity and specificity alone may bring in an inappropriate impression when comparison of the approaches adopted by different researchers is performed. This can be ascribed to wide variation size-wise and source-wise in datasets. Uniformity in algorithm performance and metrics was lacking in the suggested methods which made comparison less effective. The method adopted by Dabbah et al. [87] using multiscale dual filtering could provide an average error rate of 0.15 compared to all other single-scale and multiscale methods of segmentation. This facilitated the method to be widely accepted as a software (ACCMetrics) for clinical analysis in diabetic neuropathy. Gabor filtering method along with thresholding and SVM classifier, was utilized by Guimaraes et al. [1] achieving 90% sensitivity, low FDR and low processing time of 0.61s. Considering traditional segmentation methods, there is no doubt that methods proposed by Dabbah et al. [87] and Guimaraes et al. [1] performed well. Considering DL approaches for segmentation, method suggested by Colonna et al. [97] registered a sensitivity of 97%, and FDR of 18% with an execution time of <1s. These achievements are slightly overshadowed by the comparatively smaller number of test images used, making it less generalizable. Attention mechanisms as applied to the basic U-net, seems to have contributed to good segmentation quality, as evidenced by the works of Zhang et al. [99] and Mou et al. [100]. Wei et al. [105] could achieve 96% sensitivity, by using ResNet as the encoder in the basic U-net architecture. U-net with its modifications and advancements such as attention modules, have emerged as effective segmentation tools. But, it may be noted that U-net showed an increased tendency for FDR with added artifacts. In this regard, cGAN was found to be more robust [107].

### Fungal filaments

In traditional detection methods of dermatophytes in clinical samples, Mader et al. [111] could achieve only an accuracy of 80.3% with sensitivity and specificity of 83% and 79% respectively. Koo et al. [115] also observed that diagnosing fungal infections in clinical images using traditional methods was difficult due to the presence of several artifacts other than dermatophytes. When DL methods were used such as ResNet50 with transfer learning by Gao et al. [114], an increased sensitivity and specificity of 97% and 98% respectively were recorded. Results of the recent study using YOLOV4, indicated that application of deep learning could bring about better performance even on unstained samples.

In detection of fungal keratitis, though traditional methods could provide a performance with 99.74% accuracy [113], the method suffered from errors related to subjectivity. It is in this context that DL methods play an important role in providing good performance against this limitation.

## Conclusion

Methods for automated analysis of three curvilinear structures namely retinal blood vessels, corneal nerves and fungal filaments, which supports diagnosis of ocular and skin diseases have been described in this review. Both traditional and DL methods have been included. The observations of this review are as follows:

- In retinal blood vessel segmentation and A/V classification, in spite of significant developments, only a few methods could confront all the challenges that may crop up in an investigatory set up. Many more are warranted in this direction so as to enrich the automation process of clinical analytical procedures. A model’s robustness need to be checked by ascertaining its ability to counter the hurdles that come across in retinal blood vessel detection. It is also desirable to achieve segmentation free from fractured and broken vessels which is not the case as yet. Acceptability of a method gets enhanced when cross-validated across datasets, thus ensuring its generalization ability.
- In corneal nerve detection, a thorough comparative performance evaluation of U-net and GAN models, especially when challenged with artifacts is desirable.
- Standardization of length, density and tortuosity of corneal nerves, as also unifying the performance parameters would make the comparison more meaningful.
- There is enough potential to develop newer methods in the filamentous fungal category. Intriguing background cell lines mimicking actual dermatophyte hyphae deserves due attention. The presence of fragmented hyphae adds to the complexity of the image.
- Though the performance levels claimed by most publications for fungal detection cross 80%, validation aspects need to be updated, as there is lack of dependable publicly available datasets. Dearth of annotation in dermatophyte dataset is a glaring lacuna in its segmentation attempts.

Certain categories of curvilinear structures such as retinal blood vessels and corneal nerves, abound in publications providing good performance in automated segmentation/classification. Cross-domain adaptation of these methods to the fungal category would go a long way in avoiding the lopsidedness. Many challenges common to these curvilinear structures have been addressed by different researchers, as discussed in this review.

## Data Availability

Not applicable.

## References

[1] Guimaraes P, Wigdahl J, Ruggeri A. A fast and efficient technique for the automatic tracing of corneal nerves in confocal microscopy. Translational Vision Science & Technology. 2016;5(5). 10.1167/tvst.5.5.7 PMC5054765 27730007

[2] Bibiloni P, González-Hidalgo M, Massanet S (2016). A survey on curvilinear object segmentation in multiple applications. Pattern Recognition..

[3] Mosinska AJ. Learning approach to delineation of curvilinear structures in 2D and 3D images. EPFL; 2019.

[4] Mookiah MRK, Hogg S, MacGillivray TJ, Prathiba V, Pradeepa R, Mohan V (2021). A review of machine learning methods for retinal blood vessel segmentation and artery/vein classification. Medical Image Analysis..

[5] Mo J, Zhang L (2017). Multi-level deep supervised networks for retinal vessel segmentation. International Journal of Computer Assisted Radiology and Surgery..

[6] Oakley JD, Russakoff DB, McCarron ME, Weinberg RL, Izzi JM, Misra SL (2020). Deep learning-based analysis of macaque corneal sub-basal nerve fibers in confocal microscopy images. Eye and Vision..

[7] Haque IRI, Neubert J (2020). Deep learning approaches to biomedical image segmentation. Informatics in Medicine Unlocked..

[8] Yau JW, Rogers SL, Kawasaki R, Lamoureux EL, Kowalski JW, Bek T (2012). Global prevalence and major risk factors of diabetic retinopathy. Diabetes Care..

[9] Fraz MM, Remagnino P, Hoppe A, Uyyanonvara B, Rudnicka AR, Owen CG (2012). Blood vessel segmentation methodologies in retinal images-a survey. Computer Methods and Programs in Biomedicine..

[10] Wong TY, Klein R, Klein BE, Tielsch JM, Hubbard L, Nieto FJ (2001). Retinal microvascular abnormalities and their relationship with hypertension, cardiovascular disease, and mortality. Survey of Ophthalmology..

[11] Doubal F, Hokke P, Wardlaw J (2009). Retinal microvascular abnormalities and stroke: a systematic review. Journal of Neurology, Neurosurgery & Psychiatry..

[12] L Srinidhi C, Aparna P, Rajan J. Recent advancements in retinal vessel segmentation. Journal of Medical Systems. 2017;41(4):1-22. 10.1007/s10916-017-0719-2 28285460

[13] Chaudhuri S, Chatterjee S, Katz N, Nelson M, Goldbaum M (1989). Detection of blood vessels in retinal images using two-dimensional matched filters. IEEE Transactions on Medical Imaging..

[14] Kar SS, Maity SP (2016). Blood vessel extraction and optic disc removal using curvelet transform and kernel fuzzy c-means. Computers in Biology and Medicine..

[15] Kar SS, Maity SP. Retinal blood vessel extraction and optic disc removal using curvelet transform and morphological operation. In: Machine Intelligence and Signal Processing. Springer; 2016. p. 153-61.

[16] Kar SS, Maity SP (2016). Retinal blood vessel extraction using tunable bandpass filter and fuzzy conditional entropy. Computer Methods and Programs in Biomedicine..

[17] Tan JH, Acharya UR, Chua KC, Cheng C, Laude A (2016). Automated extraction of retinal vasculature. Medical Physics..

[18] Farokhian F, Yang C, Demirel H, Wu S, Beheshti I (2017). Automatic parameters selection of Gabor filters with the imperialism competitive algorithm with application to retinal vessel segmentation. Biocybernetics and Biomedical Engineering..

[19] Singh NP, Srivastava R (2016). Retinal blood vessels segmentation by using Gumbel probability distribution function based matched filter. Computer Methods and Programs in Biomedicine..

[20] Liu Q, Zou B, Chen J, Chen Z, Zhu C, Yue K, et al. Retinal vessel segmentation from simple to difficult. In: Proceedings of MICCAI workshop on ophthalmic medical image analysis; 2016. p. 57-64.

[21] Soomro TA, Khan MA, Gao J, Khan TM, Paul M (2017). Contrast normalization steps for increased sensitivity of a retinal image segmentation method. Signal, Image and Video Processing..

[22] Rezaee K, Haddadnia J, Tashk A (2017). Optimized clinical segmentation of retinal blood vessels by using combination of adaptive filtering, fuzzy entropy and skeletonization. Applied Soft Computing..

[23] Kovács G, Hajdu A (2016). A self-calibrating approach for the segmentation of retinal vessels by template matching and contour reconstruction. Medical Image Analysis..

[24] Imani E, Javidi M, Pourreza HR (2015). Improvement of retinal blood vessel detection using morphological component analysis. Computer Methods and Programs in Biomedicine..

[25] Dash J, Bhoi N (2017). A thresholding based technique to extract retinal blood vessels from fundus images. Future Computing and Informatics Journal..

[26] Hassan M, Amin M, Murtza I, Khan A, Chaudhry A (2017). Robust Hidden Markov Model based intelligent blood vessel detection of fundus images. Computer Methods and Programs in Biomedicine..

[27] Jiang Z, Yepez J, An S, Ko S (2017). Fast, accurate and robust retinal vessel segmentation system. Biocybernetics and Biomedical Engineering..

[28] Nergiz M, Akın M (2017). Retinal vessel segmentation via structure tensor coloring and anisotropy enhancement. Symmetry..

[29] Sazak Ç, Nelson CJ, Obara B (2019). The multiscale bowler-hat transform for blood vessel enhancement in retinal images. Pattern Recognition..

[30] Lázár I, Hajdu A (2015). Segmentation of retinal vessels by means of directional response vector similarity and region growing. Computers in Biology and Medicine..

[31] Zhang J, Chen Y, Bekkers E, Wang M, Dashtbozorg B, ter Haar Romeny BM (2017). Retinal vessel delineation using a brain-inspired wavelet transform and random forest. Pattern Recognition..

[32] Annunziata R, Garzelli A, Ballerini L, Mecocci A, Trucco E (2015). Leveraging multiscale Hessian-based enhancement with a novel exudate inpainting technique for retinal vessel segmentation. IEEE Journal of Biomedical and Health Informatics..

[33] Bahadar Khan K, A Khaliq A, Shahid M. Correction: A morphological Hessian based approach for retinal blood vessels segmentation and denoising using region based Otsu thresholding. PloS One. 2016;11(9):e0162581. 10.1371/journal.pone.0162581 PMC5008839 27583681

[34] Hassan G, Hassanien AE (2018). Retinal fundus vasculature multilevel segmentation using whale optimization algorithm. Signal, Image and Video Processing..

[35] Christodoulidis A, Hurtut T, Tahar HB, Cheriet F (2016). A multi-scale tensor voting approach for small retinal vessel segmentation in high resolution fundus images. Computerized Medical Imaging and Graphics..

[36] Pandey D, Yin X, Wang H, Zhang Y (2017). Accurate vessel segmentation using maximum entropy incorporating line detection and phase-preserving denoising. Computer Vision and Image Understanding..

[37] Rodrigues LC, Marengoni M (2017). Segmentation of optic disc and blood vessels in retinal images using wavelets, mathematical morphology and Hessian-based multi-scale filtering. Biomedical Signal Processing and Control..

[38] Zhang J, Dashtbozorg B, Bekkers E, Pluim JP, Duits R, ter Haar Romeny BM (2016). Robust retinal vessel segmentation via locally adaptive derivative frames in orientation scores. IEEE Transactions on Medical Imaging..

[39] Annunziata R, Trucco E (2016). Accelerating convolutional sparse coding for curvilinear structures segmentation by refining SCIRD-TS filter banks. IEEE Transactions on Medical Imaging..

[40] Waheed A, Akram MU, Khalid S, Waheed Z, Khan MA, Shaukat A (2015). Hybrid features and mediods classification based robust segmentation of blood vessels. Journal of Medical Systems..

[41] Ding Y, Ward WO, Duan J, Auer D, Gowland P, Bai L (2015). Retinal vasculature classification using novel multifractal features. Physics in Medicine & Biology..

[42] Tang Z, Zhang J, Gui W (2017). Selective search and intensity context based retina vessel image segmentation. Journal of Medical Systems..

[43] Barkana BD, Saricicek I, Yildirim B (2017). Performance analysis of descriptive statistical features in retinal vessel segmentation via fuzzy logic, ANN. SVM and classifier fusion. Knowledge-Based Systems..

[44] Zhu C, Zou B, Xiang Y, Cui J, Wu H (2016). An ensemble retinal vessel segmentation based on supervised learning in fundus images. Chinese Journal of Electronics..

[45] Kaur J, Mittal D (2017). A generalized method for the detection of vascular structure in pathological retinal images. Biocybernetics and Biomedical Engineering..

[46] Vega R, Sanchez-Ante G, Falcon-Morales LE, Sossa H, Guevara E (2015). Retinal vessel extraction using lattice neural networks with dendritic processing. Computers in Biology and Medicine..

[47] Aslani S, Sarnel H (2016). A new supervised retinal vessel segmentation method based on robust hybrid features. Biomedical Signal Processing and Control..

[48] Shah SAA, Tang TB, Faye I, Laude A (2017). Blood vessel segmentation in color fundus images based on regional and Hessian features. Graefe’s Archive for Clinical and Experimental Ophthalmology..

[49] Strisciuglio N, Azzopardi G, Vento M, Petkov N (2016). Supervised vessel delineation in retinal fundus images with the automatic selection of B-COSFIRE filters. Machine Vision and Applications..

[50] Oliveira WS, Teixeira JV, Ren TI, Cavalcanti GD, Sijbers J (2016). Unsupervised retinal vessel segmentation using combined filters. PloS One..

[51] Memari N, Ramli AR, Bin Saripan MI, Mashohor S, Moghbel M (2017). Supervised retinal vessel segmentation from color fundus images based on matched filtering and AdaBoost classifier. PloS One..

[52] Jebaseeli TJ, Durai CAD, Peter JD (2019). Retinal blood vessel segmentation from diabetic retinopathy images using tandem PCNN model and deep learning based SVM. Optik..

[53] Jemima Jebaseeli T, Anand Deva Durai C, Dinesh Peter J. Retinal blood vessel segmentation from depigmented diabetic retinopathy images. IETE Journal of Research. 2021;67(2):263-80.

[54] Panda R, Puhan N, Panda G (2016). New binary Hausdorff symmetry measure based seeded region growing for retinal vessel segmentation. Biocybernetics and Biomedical Engineering..

[55] Kalaie S, Gooya A (2017). Vascular tree tracking and bifurcation points detection in retinal images using a hierarchical probabilistic model. Computer Methods and Programs in Biomedicine..

[56] Srinidhi CL, Aparna P, Rajan J (2018). A visual attention guided unsupervised feature learning for robust vessel delineation in retinal images. Biomedical Signal Processing and Control..

[57] Borji A, Itti L (2012). State-of-the-art in visual attention modeling. IEEE Transactions on Pattern Analysis and Machine Intelligence..

[58] Soomro TA, Afifi AJ, Zheng L, Soomro S, Gao J, Hellwich O (2019). Deep learning models for retinal blood vessels segmentation: A review. IEEE Access..

[59] Chen C, Chuah JH, Raza A, Wang Y (2021). Retinal vessel segmentation using deep learning: A review. IEEE Access..

[60] Niemeijer M, Staal J, van Ginneken B, Loog M, Abramoff MD. Comparative study of retinal vessel segmentation methods on a new publicly available database. In: Medical imaging 2004: image processing. vol. 5370. International Society for Optics and Photonics; 2004. p. 648-56.

[61] Ronneberger O, Fischer P, Brox T. U-net: Convolutional networks for biomedical image segmentation. In: International Conference on Medical image computing and computer-assisted intervention. Springer; 2015. p. 234-41.

[62] Li K, Qi X, Luo Y, Yao Z, Zhou X, Sun M (2020). Accurate retinal vessel segmentation in color fundus images via fully attention-based networks. IEEE Journal of Biomedical and Health Informatics..

[63] Ding J, Zhang Z, Tang J, Guo F (2021). A Multichannel deep neural network for retina vessel segmentation via a fusion mechanism. Frontiers in Bioengineering and Biotechnology..

[64] Park KB, Choi SH, Lee JY (2020). M-GAN: Retinal blood vessel segmentation by balancing losses through stacked deep fully convolutional networks. IEEE Access..

[65] Li X, Jiang Y, Rodriguez-Andina JJ, Luo H, Yin S, Kaynak O (2021). When medical images meet generative adversarial network: recent development and research opportunities. Discover Artificial Intelligence..

[66] Grisan E, Foracchia M, Ruggeri A (2008). A novel method for the automatic grading of retinal vessel tortuosity. IEEE Transactions on Medical Imaging..

[67] Yan Y, Wen D, Dewan M, Huang WB. Classification of artery and vein in retinal fundus images based on the context-dependent features. In: International Conference on Digital Human Modeling and Applications in Health, Safety, Ergonomics and Risk Management. Springer; 2017. p. 198-213.

[68] Xu X, Ding W, Abràmoff MD, Cao R (2017). An improved arteriovenous classification method for the early diagnostics of various diseases in retinal image. Computer Methods and Programs in Biomedicine..

[69] Zhu C, Zou B, Zhao R, Cui J, Duan X, Chen Z (2017). Retinal vessel segmentation in colour fundus images using extreme learning machine. Computerized Medical Imaging and Graphics..

[70] Akbar S, Akram MU, Sharif M, Tariq A, ullah Yasin U. Arteriovenous ratio and papilledema based hybrid decision support system for detection and grading of hypertensive retinopathy. Computer Methods and Programs in Biomedicine. 2018;154:123-41. 10.1016/j.cmpb.2017.11.014 29249337

[71] Yin X, Irshad S, Zhang Y (2020). Classifiers fusion for improved vessel recognition with application in quantification of generalized arteriolar narrowing. Journal of Innovative Optical Health Sciences..

[72] Vijayakumar V, Koozekanani DD, White R, Kohler J, Roychowdhury S, Parhi KK. Artery/vein classification of retinal blood vessels using feature selection. In: 2016 38th annual international conference of the IEEE Engineering in Medicine and Biology Society (EMBC). IEEE; 2016. p. 1320-3. 10.1109/EMBC.2016.7590950 28268568

[73] Pellegrini E, Robertson G, MacGillivray T, van Hemert J, Houston G, Trucco E (2017). A graph cut approach to artery/vein classification in ultra-widefield scanning laser ophthalmoscopy. IEEE Transactions on Medical Imaging..

[74] Srinidhi CL, Aparna P, Rajan J (2019). Automated method for retinal artery/vein separation via graph search metaheuristic approach. IEEE Transactions on Image Processing..

[75] Zhao Y, Xie J, Zhang H, Zheng Y, Zhao Y, Qi H (2019). Retinal vascular network topology reconstruction and artery/vein classification via dominant set clustering. IEEE Transactions on Medical Imaging..

[76] Girard F, Cheriet F. Artery/vein classification in fundus images using CNN and likelihood score propagation. In: 2017 IEEE Global Conference on Signal and Information Processing (GlobalSIP). IEEE; 2017. p. 720-4.

[77] Girard F, Kavalec C, Cheriet F (2019). Joint segmentation and classification of retinal arteries/veins from fundus images. Artificial Intelligence in Medicine..

[78] Xu X, Tan T, Xu F. An improved U-net architecture for simultaneous arteriole and venule segmentation in fundus image. In: Annual Conference on Medical Image Understanding and Analysis. Springer; 2018. p. 333-40.

[79] Hemelings R, Elen B, Stalmans I, Van Keer K, De Boever P, Blaschko MB (2019). Artery-vein segmentation in fundus images using a fully convolutional network. Computerized Medical Imaging and Graphics..

[80] Ma W, Yu S, Ma K, Wang J, Ding X, Zheng Y. Multi-task neural networks with spatial activation for retinal vessel segmentation and artery/vein classification. In: International Conference on Medical Image Computing and Computer-Assisted Intervention. Springer; 2019. p. 769-78.

[81] Yang J, Dong X, Hu Y, Peng Q, Tao G, Ou Y (2020). Fully automatic arteriovenous segmentation in retinal images via topology-aware generative adversarial networks. Interdisciplinary Sciences: Computational Life Sciences..

[82] Hu J, Wang H, Cao Z, Wu G, Jonas JB, Wang YX, et al. Automatic artery/vein classification using a vessel-constraint network for multicenter fundus images. Frontiers in Cell and Developmental Biology. 2021;9. 10.3389/fcell.2021.659941 PMC8226261 34178986

[83] Kim KM, Son K, Palmore GTR (2015). Neuron image analyzer: Automated and accurate extraction of neuronal data from low quality images. Scientific Reports..

[84] Salahuddin T, Qidwai U (2020). Computational methods for automated analysis of corneal nerve images: Lessons learned from retinal fundus image analysis. Computers in Biology and Medicine..

[85] Herrera-Pereda R, Crispi AT, Babin D, Philips W, Costa MH (2021). A Review on digital image processing techniques for in-vivo confocal images of the cornea. Medical Image Analysis..

[86] Petropoulos IN, Ponirakis G, Khan A, Gad H, Almuhannadi H, Brines M (2020). Corneal confocal microscopy: ready for prime time. Clinical and Experimental Optometry..

[87] Dabbah MA, Graham J, Petropoulos IN, Tavakoli M, Malik RA (2011). Automatic analysis of diabetic peripheral neuropathy using multi-scale quantitative morphology of nerve fibres in corneal confocal microscopy imaging. Medical Image Analysis..

[88] Scarpa F, Grisan E, Ruggeri A (2008). Automatic recognition of corneal nerve structures in images from confocal microscopy. Investigative Ophthalmology & Visual Science..

[89] Tavakoli M, Ferdousi M, Petropoulos IN, Morris J, Pritchard N, Zhivov A (2015). Normative values for corneal nerve morphology assessed using corneal confocal microscopy: a multinational normative data set. Diabetes Care..

[90] Annunziata R, Kheirkhah A, Aggarwal S, Hamrah P, Trucco E (2016). A fully automated tortuosity quantification system with application to corneal nerve fibres in confocal microscopy images. Medical Image Analysis..

[91] Al-Fahdawi S, Qahwaji R, Al-Waisy AS, Ipson S, Malik RA, Brahma A (2016). A fully automatic nerve segmentation and morphometric parameter quantification system for early diagnosis of diabetic neuropathy in corneal images. Computer Methods and Programs in Biomedicine..

[92] Hosseinaee Z, Tan B, Kralj O, Han L, Wong A, Sorbara L, et al. Fully automated corneal nerve segmentation algorithm for corneal nerves analysis from in-vivo UHR-OCT images. In: Ophthalmic Technologies XXIX. vol. 10858. International Society for Optics and Photonics; 2019. p. 1085823.

[93] Silva SF, Gouveia S, Gomes L, Negrão L, Quadrado MJ, Domingues JP, et al. Diabetic peripheral neuropathy assessment through texture based analysis of corneal nerve images. In: Journal of Physics: Conference Series. vol. 616. IOP Publishing; 2015. p. 012002.

[94] Salahuddin T, Qidwai U. Classification of corneal nerve images using machine learning techniques. International Journal of Integrated Engineering. 2019;11.

[95] Litjens G, Kooi T, Bejnordi BE, Setio AAA, Ciompi F, Ghafoorian M (2017). A survey on deep learning in medical image analysis. Medical Image Analysis..

[96] Ibtehaz N, Rahman MS (2020). MultiResUNet: Rethinking the U-Net architecture for multimodal biomedical image segmentation. Neural Networks..

[97] Colonna A, Scarpa F, Ruggeri A. Segmentation of corneal nerves using a U-net-based convolutional neural network. In: Computational Pathology and Ophthalmic Medical Image Analysis. Springer; 2018. p. 185-92.

[98] Williams BM, Borroni D, Liu R, Zhao Y, Zhang J, Lim J (2020). An artificial intelligence-based deep learning algorithm for the diagnosis of diabetic neuropathy using corneal confocal microscopy: a development and validation study. Diabetologia..

[99] Zhang D, Huang F, Khansari M, Berendschot TT, Xu X, Dashtbozorg B (2020). Automatic corneal nerve fiber segmentation and geometric biomarker quantification. The European Physical Journal Plus..

[100] Mou L, Zhao Y, Fu H, Liu Y, Cheng J, Zheng Y (2021). CS2-Net: Deep learning segmentation of curvilinear structures in medical imaging. Medical Image Analysis..

[101] Zhang Z, Liu Q, Wang Y (2018). Road extraction by deep residual U-net. IEEE Geoscience and Remote Sensing Letters..

[102] Zhou Z, Rahman Siddiquee MM, Tajbakhsh N, Liang J. Unet++: A nested U-net architecture for medical image segmentation. In: Deep learning in medical image analysis and multimodal learning for clinical decision support. Springer; 2018. p. 3-11. 10.1007/978-3-030-00889-5_1 PMC7329239 32613207

[103] Oktay O, Schlemper J, Folgoc LL, Lee M, Heinrich M, Misawa K, et al. Attention U-net: Learning where to look for the pancreas. arXiv preprint arXiv:1804.03999. 2018.

[104] Fu J, Liu J, Tian H, Li Y, Bao Y, Fang Z, et al. Dual attention network for scene segmentation. In: Proceedings of the IEEE/CVF conference on computer vision and pattern recognition; 2019. p. 3146-54.

[105] Wei S, Shi F, Wang Y, Chou Y, Li X (2020). A deep learning model for automated sub-basal corneal nerve segmentation and evaluation using in vivo confocal microscopy. Translational Vision Science & Technology..

[106] Lin L, Cheng P, Wang Z, Li M, Wang K, Tang X. Automated segmentation of corneal nerves in confocal microscopy via contrastive learning based synthesis and quality enhancement. In: 2021 IEEE 18th International Symposium on Biomedical Imaging (ISBI). IEEE; 2021. p. 1314-8.

[107] Yıldız E, Arslan AT, Taş AY, Acer AF, Demir S, Şahin A (2021). Generative Adversarial Network based automatic segmentation of corneal subbasal nerves on in vivo confocal microscopy images. Translational Vision Science & Technology..

[108] Isola P, Zhu JY, Zhou T, Efros AA. Image-to-image translation with conditional adversarial networks. In: Proceedings of the IEEE conference on computer vision and pattern recognition; 2017. p. 1125-34.

[109] Mirza M, Osindero S. Conditional generative adversarial nets. arXiv preprint arXiv:1411.1784. 2014.

[110] Reichl U, Yang H, Gilles ED, Wolf H (1990). An improved method for measuring the interseptal spacing in hyphae of Streptomyces tendae by fluorescence microscopy coupled with image processing. FEMS Microbiology Letters..

[111] Mäder U, Quiskamp N, Wildenhain S, Schmidts T, Mayser P, Runkel F, et al. Image-processing scheme to detect superficial fungal infections of the skin. Computational and Mathematical Methods in Medicine. 2015;2015. 10.1155/2015/851014 PMC4663297 26649072

[112] Qiu Q, Liu Z, Zhao Y, Wei D, Wu X. Automatic detecting cornea fungi based on texture analysis. In: 2016 IEEE International Conference on Smart Cloud (SmartCloud). IEEE; 2016. p. 214-7.

[113] Wu X, Qiu Q, Liu Z, Zhao Y, Zhang B, Zhang Y (2018). Hyphae detection in fungal keratitis images with adaptive robust binary pattern. IEEE Access..

[114] Gao W, Li M, Wu R, Du W, Zhang S, Yin S (2021). The design and application of an automated microscope developed based on deep learning for fungal detection in dermatology. Mycoses..

[115] Koo T, Kim MH, Jue MS (2021). Automated detection of superficial fungal infections from microscopic images through a regional convolutional neural network. PloS One..

[116] Sopo CJP, Hajati F, Gheisari S. DeFungi: Direct mycological examination of microscopic fungi images. arXiv preprint arXiv:2109.07322. 2021.

[117] Rajitha KV, Sowmya B, Prakash PY, Raghavendra R, Keerthana P. Classification of microscopic images of unstained skin samples using deep learning approach. In: 2022 IEEE 19th International Symposium on Biomedical Imaging (ISBI). IEEE; 2022. .

[118] Wu X, Tao Y, Qiu Q, Wu X (2018). Application of image recognition-based automatic hyphae detection in fungal keratitis. Australasian Physical & Engineering Sciences in Medicine..

[119] Lv J, Zhang K, Chen Q, Chen Q, Huang W, Cui L, et al. Deep learning-based automated diagnosis of fungal keratitis with in vivo confocal microscopy images. Annals of Translational Medicine. 2020;8(11). 10.21037/atm.2020.03.134 PMC7327373 32617326

[120] Liu Z, Cao Y, Li Y, Xiao X, Qiu Q, Yang M (2020). Automatic diagnosis of fungal keratitis using data augmentation and image fusion with deep convolutional neural network. Computer Methods and Programs in Biomedicine..

[121] He K, Zhang X, Ren S, Sun J. Deep residual learning for image recognition. In: Proceedings of the IEEE conference on computer vision and pattern recognition; 2016. p. 770-8.

[122] Yu F, Koltun V. Multi-scale context aggregation by dilated convolutions. arXiv preprint arXiv:1511.07122. 2015.

[123] Vaswani A, Shazeer N, Parmar N, Uszkoreit J, Jones L, Gomez AN, et al. Attention is all you need. Advances in Neural Information Processing Systems. 2017;30.

